# The Impact of 12‐Hour Versus 8‐Hour Shifts on Intensive Care Unit Nurses’ Physical and Mental Well‐Being and Job Satisfaction: A Systematic Review

**DOI:** 10.1155/jonm/3524359

**Published:** 2026-10-28

**Authors:** Ammar Othman, Danielle Najm, Edward Zimbudzi

**Affiliations:** ^1^ College of Nursing in Khamis Mushait King Khalid University Abha Asir Saudi Arabia kku.edu.sa; ^2^ School of Nursing and Midwifery Faculty of Medicine, Nursing and Health Sciences Monash University Melbourne Victoria Australia monash.ac.za

**Keywords:** 8-h shifts, 12-h shifts, job satisfaction, physical and mental health

## Abstract

**Background:**

Twelve‐hour shifts have been widely implemented in intensive care units (ICUs), where nurses work in high‐acuity environments requiring sustained clinical attention. However, evidence regarding the impact of shift length on ICU nurses’ physical health, mental well‐being, job satisfaction and patient‐care outcomes remains limited and inconsistent.

**Objective:**

To investigate the impact of 12‐h compared with 8‐h shifts on ICU nurses’ physical health, well‐being and job satisfaction.

**Design:**

This systematic review was conducted in accordance with the Preferred Reporting Items for Systematic Reviews and Meta‐Analyses guidelines and was registered with PROSPERO (CRD420251021178).

**Methods:**

A systematic search of CINAHL, Ovid MEDLINE and Embase was conducted in February 2025 and updated in June 2026. Additional searches of Scopus and Web of Science were undertaken in June 2026. Eligible studies included ICU nurses and compared 12‐h with 8‐h shifts. Risk of bias and methodological quality were assessed using design‐specific Joanna Briggs Institute (JBI) critical appraisal tools and the Mixed Methods Appraisal Tool (MMAT). Certainty of evidence was assessed using the Grading of Recommendations Assessment, Development and Evaluation approach. Findings were synthesised narratively due to the heterogeneity of the included studies.

**Result:**

Of 815 records identified, five studies met the inclusion criteria. Findings on fatigue, sleep and burnout were inconclusive across studies. Twelve‐hour shifts were generally associated with greater roster satisfaction, flexibility and work–life balance, while evidence for sick leave was limited or mixed.

**Conclusion:**

Evidence did not consistently favour either 12‐ or 8‐h shifts for ICU nurses, with low to very low certainty of evidence. Shift duration should therefore be considered alongside overall roster design, staffing needs and nurses’ well‐being when planning ICU schedules.

**Implications for Nursing Management:**

Nurse managers should avoid a one‐size‐fits‐all approach to shift duration, as evidence does not clearly favour 12‐ or 8‐h shifts. Shift‐length decisions should consider roster design, staffing needs, workload, recovery time and nurses’ preferences. Managers should also monitor staff well‐being, satisfaction, absenteeism, retention and patient‐care outcomes when reviewing shift patterns. Given the low certainty of evidence, ongoing local evaluation is essential to ensure rosters support workforce sustainability and safe, high‐quality care.

## 1. Background

Intensive care units (ICUs) are high‐acuity environments characterised by complex care delivery, continuous patient surveillance and substantial workload demands [1, 2]. ICU nursing requires sustained vigilance and safety‐critical decision‐making throughout every shift [3]. In this context, shift duration may have important implications for nurses’ physical health, well‐being and job satisfaction as well as for the safe and effective delivery of patient care.

Twelve‐hour shifts have been widely implemented in healthcare settings to address staffing and scheduling requirements [4, 5]. Potential advantages include fewer working days and fewer patient handovers, which are often perceived as supporting continuity of care, as well as greater scheduling flexibility [6]. Changes in shift schedules can also influence nurses’ work–life balance and satisfaction with scheduling [6, 7]. Longer shifts have been associated with increased fatigue. Chen et al. [8] found that shift schedules, work–family conflict, compensatory sleep and daytime sleepiness were associated with fatigue among ICU nurses. Job satisfaction is also influenced by multiple factors, including the work environment, workload, emotional exhaustion and interprofessional relationships, with shift length representing another potentially important modifiable factor [9, 10].

Evidence comparing 8‐ and 12‐h shifts has produced inconsistent findings [5]. Iskera‐Golec et al. [11] found that ICU nurses working 12‐h shifts experienced higher levels of chronic fatigue, cognitive anxiety, sleep disturbance and emotional exhaustion than those working 8‐h shifts. However, Battle and Temblett [12] reported lower emotional exhaustion and depersonalisation following the introduction of a 12‐h roster, with no significant difference in sickness rates between the 8‐ and 12‐h roster periods. These differing findings highlight uncertainty regarding the relationship between shift duration and nurses’ physical health and well‐being.

Previous reviews of 12‐h nursing shifts have widely examined nurses across general hospital settings [5, 13]. However, evidence directly comparing 12‐ and 8‐h shift patterns among ICU nurses remains limited and inconsistent. The novelty of this review lies in its exclusive focus on ICU nurses and its integrated examination of multiple nurse and patient‐care outcomes within a single synthesis. Accordingly, this systematic review aimed to examine the impact of 12‐h compared with 8‐h shifts on ICU nurses’ physical health, well‐being and job satisfaction, providing a contemporary synthesis of evidence relevant to ICU workforce and roster management outcomes.

### 1.1. Methods and Design

The review aligned with Preferred Reporting Items for Systematic Reviews and Meta‐Analyses (PRISMA) guidelines to ensure methodological rigour and transparency [14]. The review was registered with PROSPERRO (Registration ID: CRD420251021178). This systematic review utilised the population, intervention, comparison, outcomes (PICOs) framework to ensure a structured and transparent approach to evaluate the impact of extended working hours on nursing outcomes (Table 1).

**TABLE 1:** Selection criteria.

	Inclusion	Exclusion
Participants	Nurses who worked in intensive care units	Studies focused exclusively on other healthcare professionals or studies where data could not be attributed to nurses (i.e., the data were presented as a whole), were excluded.
Interventions	The intervention of interest was 12‐h nursing shifts, defined as routinely scheduled shifts of approximately 12 h’ duration.	Interventions that were not focused on extended working hours (such as studies unrelated to shifts exceeding 8 h).
Control	The comparator included 8‐h shifts, defined as scheduled nursing shifts of approximately 8 h in duration.	Studies that did not include standard working hours (shifts lasting 8 h or less) as a comparator.
Outcomes	Measures related to nurses’ health (stress, physical ailments, fatigue, sleep quality), well‐being (burnout, stress) and job satisfaction (work–life balance, intention to leave the profession, attitudes towards 12‐h shifts).	
Studies	Studies of any design (quantitative, qualitative, or mixed methods) that focused on contemporary working conditions. Consideration of cultural and regional differences in the impact of 12‐h shifts.	Studies published in languages other than English or with significant ethical issues.

### 1.2. Participants

The review focused on nurses working in ICUs, including registered nurses and licensed practical nurses, without restrictions on geographical location, age, or gender.

### 1.3. Intervention

The intervention of interest was 12‐h shifts, defined as scheduled work shifts of approximately 12 h in duration. The comparator was 8‐h shifts, defined as scheduled work shifts of approximately 8 h in duration. Studies comparing these shift durations within ICU nursing populations were eligible for inclusion.

### 1.4. Outcomes

Primary outcomes included all physical and psychological symptoms, specifically fatigue, sleep disturbances, psychological stress and burnout. Secondary outcomes included well‐being–related measures (for example, work–life balance) and job satisfaction indicators, including retention‐related outcomes (for example, turnover or intention to leave).

### 1.5. Studies

Eligible studies included quantitative, qualitative and mixed‐methods primary research that examined the impact of 12‐h compared with 8‐h shifts among ICU nurses.

### 1.6. Search Strategy

Key databases were searched, including CINAHL for its emphasis on nursing and allied health literature, Ovid MEDLINE for its comprehensive biomedical coverage, Embase for its broad international scope in biomedical and healthcare research, Scopus for its extensive multidisciplinary coverage and Web of Science for its broad multidisciplinary coverage and citation tracking capabilities. A systematic search of CINAHL, Ovid MEDLINE and Embase was conducted in February 2025 and updated in June 2026. Additional searches of Scopus and Web of Science were undertaken in June 2026. Appendix A (Tables A1, A2, A3, A4 and A5) summarises the search strategy.

### 1.7. Study Screening and Selection

Records from the databases were imported into EndNote 21 for deduplication. Duplicate records were removed using the software’s deduplication settings, followed by manual checking. The deduplicated library was then imported into Covidence for screening. Titles and abstracts were screened independently by the review team members (3 reviewers). Conflicts were resolved through consensus during scheduled meetings. Full‐text articles were also screened independently, with disagreements resolved through discussion and agreement (Table 2).

**TABLE 2:** Characteristics of included studies.

Author, country	Method; participants; setting	Intervention/comparator	Outcome measures	Key results	Notes
Battle and Temblett [12], United Kingdom (Wales)	Prospective service evaluation conducted over 2 years in a 28‐bed tertiary ICU. Around 150 nurses participated during the study period.	8‐h roster period vs. 12‐h roster period	Burnout (Maslach Burnout Inventory subscales). Other measures included clinical incidents, sickness rates, personal injuries and staff training.	Emotional exhaustion and depersonalisation decreased following transition to 12‐h shifts (*p* < 0.05); personal accomplishment unchanged; routinely collected indicators reported as stable.	Burnout survey response rates were 40% and 42%. During the 12‐h period, some nurses continued to work 8‐h shifts.
Dwyer et al. [19], Australia	Descriptive exploratory study conducted in a six‐bed regional ICU. Nineteen staff responded overall. The main quantitative findings were based on 12 ICU nurses working the 12‐h roster.	12‐h roster trial compared with prior 8‐h roster	Purpose‐designed questionnaires assessed sleep, fatigue, recovery, physical and psychological well‐being, work satisfaction, roster satisfaction and perceived sick leave.	Nurses reported less tiredness at work, more time to recover between shifts and improvements in physical and psychological well‐being. Roster satisfaction was high.	Perception‐focused outcomes; objective turnover low/limited reporting.
Iskera‐Golec et al. [11], Poland	Observational comparative study of ICU nurses working different roster lengths	12‐h shifts vs. 8‐h shifts	Health and well‐being indicators; fatigue; burnout (Maslach Burnout Inventory dimensions)	Nurses on 12‐h shifts reported greater chronic fatigue; emotional exhaustion was higher in 12‐h nurses; other burnout dimensions showed no clear differences.	Observational design; potential confounding by workload and scheduling context.
Ito‐Masui et al. [15], Japan	A pre–post observational study conducted in a 26‐bed university hospital ICU. Eighty nurses were included. Thirty‐seven were in the 12‐h group and 43 were in the 8‐h group. Fifteen nurses participated under both schedules.	A rotating 12‐h schedule was compared with a rotating 8‐h schedule.	Sleep was measured using Fitbit wearable devices. Other outcomes included daily well‐being, perceived stress, fatigue, sleep efficiency, sleep episodes and sleep regularity.	Total time in bed did not differ significantly between groups. Morning and evening well‐being were higher in the 12‐h group, but the differences were not significant after adjustment. Stress and fatigue were also lower in the 12‐h group, but not significantly after adjustment.	Nurses on the 12‐h schedule had longer intervals between shifts and more days off. These differences make it difficult to separate the effect of shift length from the wider roster structure.
Webster et al. [16], Australia	Service evaluation (before/after roster change) with staff surveys and routinely collected data; ICU nurses; two survey periods (8‐h vs. 12‐h)	8‐h roster period vs. 12‐h roster period (reintroduction)	Roster satisfaction; perceived work–life balance; organisational indicators (e.g., adverse event register, leave measures)	Staff satisfaction increased after the reintroduction of 12‐h shifts; routine safety indicators reported as stable; turnover low in both roster periods.	Administrative incident systems may capture different aspects of safety than self‐report.

### 1.8. Data Abstraction

Data were extracted in Covidence using a standardised abstraction form. Following extraction, team meetings were held to review extracted data, resolve discrepancies and ensure consistency and accuracy, consequently minimising extraction bias.

### 1.9. Quality Assessment

The methodological quality of all included studies was critically appraised using tools appropriate to each study design. Battle and Temblett [12], Ito‐Masui et al. [15] and Webster et al. [16], were assessed using the Joanna Briggs Institute (JBI) Critical Appraisal Tool for Quasi‐Experimental Studies [17]. Iskera‐Golec et al. [11] was assessed using the JBI Critical Appraisal Tool for Analytical Cross‐Sectional Studies [18]. Dwyer et al. [19] was assessed using the Mixed Methods Appraisal Tool (MMAT) [20]. The quality assessment of each included study was undertaken by consensus among the three reviewers, with any differences resolved through discussion until agreement was reached. No automation tools were used in the quality assessment process. These tools were selected because they provide design‐specific criteria for assessing methodological quality. The methodological strengths and limitations of each study were considered when interpreting its findings. No studies were excluded following quality assessment.

### 1.10. Data Synthesis

A narrative synthesis was undertaken as the included studies were heterogeneous in design, roster characteristics, participant characteristics, outcome measures and statistical reporting. Meta‐analysis was considered but was not feasible because too few studies reported comparable outcomes, and insufficient quantitative data were available for meaningful statistical pooling.

Following data extraction, one reviewer coded the study findings, which was informed by established guidance on narrative synthesis [21]. The coding process and completed coding were independently reviewed by the other reviewers, with discrepancies discussed and resolved by consensus. Coding was guided by the three prespecified review domains of health, well‐being and job satisfaction. Relevant findings from each study were coded according to the outcome assessed, measurement approach, direction of the finding and reported statistical significance.

Measurement approaches were differentiated as objective measures, administrative records, validated self‐report measures and purpose‐designed or perception‐based measures. Similar terminology referring to the same outcome was grouped together where appropriate, such as fatigue and tiredness. However, outcomes measured using validated tools were kept separate from those based on participants’ perceptions. The resulting outcomes were then organised within the corresponding prespecified review domain. Potential sources of heterogeneity were examined after completion of the coding process as part of the narrative synthesis.

Following coding, findings were organised within the three prespecified review domains of health, well‐being and job satisfaction and subsequently grouped by outcome. For each outcome, findings were compared according to the direction of the result and reported statistical significance. The type of measurement was also considered by distinguishing objective measures and administrative records, validated self‐report measures and purpose‐designed or perception‐based measures. Methodological considerations identified through the quality assessment were considered when synthesising and interpreting the findings.

Heterogeneity was explored descriptively after the outcome‐level synthesis by comparing study design, roster characteristics, participant characteristics, measurement approaches and study context across the included studies. These comparisons were used to identify potential sources of variation in findings. No formal subgroup analysis was undertaken.

### 1.11. Certainty of Evidence

The certainty of evidence for each synthesised outcome was assessed using the Grading of Recommendations Assessment, Development and Evaluation (GRADE) approach [22]. As all included studies were nonrandomised, the initial certainty rating was low. For each outcome, the evidence was evaluated for risk of bias, inconsistency, indirectness, imprecision and reporting bias. Risk‐of‐bias judgements were informed by the completed critical appraisals of the included studies. As meta‐analysis was not undertaken, these domains were considered based on the methodological limitations of the contributing studies, the consistency of findings, their relevance to the review question, the precision of the available evidence and the possibility of reporting bias. Imprecision was judged according to sample size, the availability of effect estimates and confidence intervals, and the adequacy of statistical reporting, with outcomes supported by only one or two studies considered at greater risk of imprecision. Publication bias was considered qualitatively because formal assessment was not possible owing to the small evidence base and lack of quantitative synthesis. Overall certainty was classified as high, moderate, low or very low.

## 2. Results

The database searches identified 815 records, including 138 from CINAHL, 133 from Ovid MEDLINE, 143 from Embase, 244 from Scopus and 157 from Web of Science. After 209 duplicates were removed in EndNote, 606 records were imported into Covidence. A further 82 duplicates were identified and removed in Covidence, resulting in 524 records for title and abstract screening. At this stage, 443 records were excluded. The remaining 81 articles underwent full‐text assessment, of which 76 were excluded. Five studies were ultimately included in the review (Figure 1).

**FIGURE 1:**
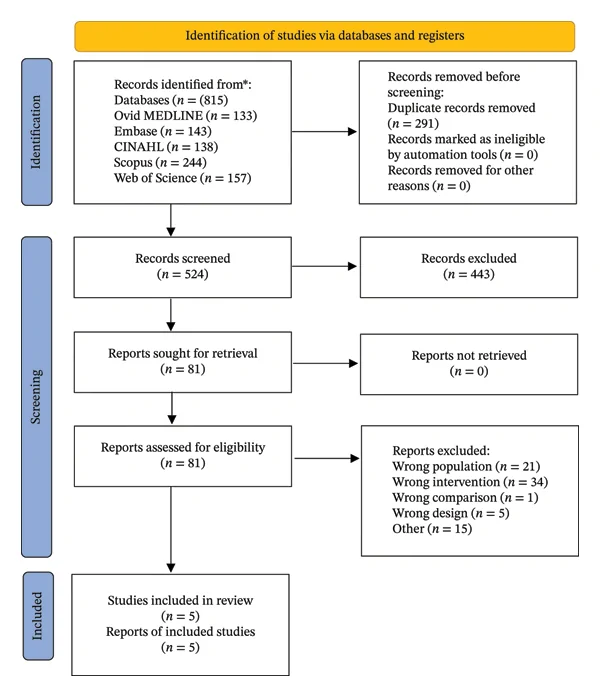
PRISMA flow diagram for studies screened.

### 2.1. Characteristics of Included Studies

Five studies included in the review. Two were conducted in Australia [16, 19], while the remaining studies were undertaken in Wales [12], Poland [11] and Japan [15]. All studies involved nurses working in ICU settings and examined outcomes associated with 12‐ and 8‐h shift patterns. The studies varied in design and included a service evaluation [12], a descriptive exploratory study [19], an analytical cross‐sectional study by Iskeragolec et al. [11], a pre–post observational study [15] and a two‐phase survey [16]. The nature of the comparison also differed across studies. One study directly compared groups of nurses working 12‐h and 8‐h shifts (Iskera‐Golec et al. [11]), whereas others assessed outcomes before and after a roster change [12, 15, 16] or asked participants to compare their experiences of the two shift patterns [19]. Outcomes were measured using validated instruments [11, 12, 15], wearable devices [15], organisational records [12, 16] and purpose‐designed or perception‐based questionnaires [16, 19]. These outcomes covered health, well‐being, job satisfaction and selected organisational and patient safety measures (Table 2).

### 2.2. Quality of Included Studies

All five included studies underwent critical appraisal, and none were excluded following quality assessment. The appraisal identified several methodological strengths, including the use of validated instruments [11, 12], objective or routinely collected organisational data [12, 16], matched comparison groups Iskera‐Golec et al. [11] and repeated measurements using wearable sensors [15].

Several methodological limitations were also noted. These included the absence of concurrent control groups in some studies [12, 15, 16, 19], potential confounding [12, 15], small sample sizes [12, 15, 19], and low or reduced response rates for some survey outcomes [12, 16, 19]. Additionally, several studies relied on perception‐based measures for some outcomes [16, 19]. Detailed appraisal findings are presented in Tables 3, 4 and 5.

**TABLE 3:** JBI critical appraisal of the included quasi experimental studies.

Criterion	Appraisal question	Battle and Temblett [12]	Ito‐Masui et al. [15]	Webster et al. [16]
Q1	Is it clear in the study what is the ‘cause’ and what is the ‘effect’ (i.e., there is no confusion about which variable comes first)?	Y	Y	Y

Q2	Was there a control group?	N	Y	N

Q3	Were participants included in any comparisons similar?	U	N	N

Q4	Were the participants included in any comparisons receiving similar treatment/care, other than the exposure or intervention of interest?	U	Y	Y

Q5	Were there multiple measurements of the outcome, both pre‐ and postintervention/exposure?	Y: clinical incidents, sickness rates, personal injuries, staff training N: emotional exhaustion, depersonalisation, personal accomplishment	Y	N

Q6	Were the outcomes of participants included in any comparisons measured in the same way?	Y	Y	Y

Q7	Were outcomes measured in a reliable way?	Y	Y: wearable sleep outcomes U: well‐being, stress, fatigue, subjective sleep quality	Y: sick/family leave U: job satisfaction, perceived health, sleep, work–life balance, fatigue

Q8	Was follow‐up complete, and if not, were differences between groups in terms of their follow‐up adequately described and analysed?	Y	Y	Y: sick/family leave N: job satisfaction, perceived health, sleep, work–life balance. Fatigue

Q9	Was appropriate statistical analysis used?	U: clinical incidents, sickness rates, staff training Y: emotional exhaustion, depersonalisation, personal accomplishment N: personal injuries	Y: total time in bed, sleep efficiency, morning/evening well‐being, subjective sleep quality U: sleep episodes/day, stress, fatigue	N: job satisfaction, perceived health U: sick/family leave, work–life balance Y: sleep, fatigue

	Overall appraisal/review decision	Include	Include	Include

*Note:* Y = yes. N = no. U = unclear. For JBI criteria assessed at the outcome or result level, the relevant official responses are reported by outcome within each study cell.

**TABLE 4:** JBI critical appraisal of the analytical cross‐sectional study.

Criterion	Question appraisal	Iskera‐Golec et al. [11]
Q1	Were the criteria for inclusion in the sample clearly defined?	Y
Q2	Were objective, standard criteria used for measurement of the condition?	N/A
Q3	Was the exposure measured in a valid and reliable way?	Y
Q4	Were the outcomes measured in a valid and reliable way?	Y
Q5	Were confounding factors identified?	Y
Q6	Were strategies to deal with confounding factors stated?	Y
Q7	Was appropriate statistical analysis used?	U
Q8	Were the study subjects and the setting described in detail?	Y
	Overall appraisal/review decision	Include

Note: Y = yes. U = unclear. N/A = not applicable. Q3 was resolved as yes because the study explicitly defined exposure as 12‐h or 8‐h shift schedules and described shift times, rotation patterns and monthly working hours.

**TABLE 5:** MMAT appraisal of the mixed‐methods study.

Criterion	Question appraisal	Dwyer et al. [19]
5.1	Is there an adequate rationale for using a mixed methods design to address the research question?	Y
5.2	Are the different components of the study effectively integrated to answer the research question?	Y
5.3	Are the outputs of the integration of qualitative and quantitative components adequately interpreted?	Y
5.4	Are divergences and inconsistencies between quantitative and qualitative results adequately addressed?	N
5.5	Do the different components of the study adhere to the quality criteria of each tradition of the methods involved?	N
	Review decision	Include

*Note:* Y = yes. N = no. The MMAT discourages calculation of an overall numerical score from individual criteria. Accordingly, no percentage or total quality score is reported.

### 2.3. Narrative Synthesis With Embedded Stratified Interpretation

Results are presented by outcome within the three prespecified domains. For numerical outcomes, study‐reported means and standard deviations or medians were presented when available. Categorical outcomes were summarised using proportions or percentages. Effect estimates, including risk ratios with 95% confidence intervals and *p* values, were reported when provided by the original studies. Because statistical pooling was not performed, no common effect measure was calculated.

### 2.4. Health

#### 2.4.1. Fatigue

Findings for fatigue were inconsistent and differed according to the way fatigue was measured. Using the Standard Shiftwork Index (SSI), Iskera‐Golec et al. [11] found significantly higher chronic fatigue among nurses working 12‐h shifts compared to those working 8‐h shifts (26.97 vs. 23.75, *p* < 0.01). In contrast, perception‐based findings generally indicated a preference for the 12‐h roster. Webster et al. [16] reported that 81% (*n* = 216) of respondents perceived fatigue as more manageable with a 12‐h roster, compared with 3.8% (*n* = 10) who perceived it as more manageable with an 8‐h roster. Similarly, Dwyer et al. [19] found that participants generally disagreed that they experienced greater fatigue at work under the 12‐h roster (*M* = 2.33, SD = 1.07) and reported greater opportunities for recovery between shifts (*M* = 3.83, SD = 0.83). Ito‐Masui et al. [15] reported lower daily self‐rated fatigue in the 12‐h cohort. However, the difference was not statistically significant after adjustment for multiple comparisons (adjusted *p* = 0.31).

### 2.5. Sleep

Findings for sleep‐related outcomes were inconsistent across the four studies reporting this outcome [11, 15, 16, 19]. Using sleep‐related measures from the SSI to assess sleep disturbance and tiredness after sleep, Iskera‐Golec et al. [11] found greater general sleep disturbance among nurses working 12‐h shifts (12.50 vs. 10.00, *p* < 0.01), as well as greater tiredness after sleep (13.36 vs. 11.38, *p* < 0.05). Average sleep duration did not differ significantly between groups (6.11 vs. 5.43 h).

Perception‐based findings generally indicated more favourable perceptions of sleep under the 12‐h roster. Webster et al. [16] reported that the proportion of nurses indicating that they obtained ‘plenty’ or ‘enough’ sleep increased from 21.1% to 89.5% after the introduction of the 12‐h roster (*p* < 0.0001). Additionally, Dwyer et al. [19] reported that participants rated the amount of sleep more favourably under the 12‐h roster (3.5 vs. 2.7, *p* = 0.03). However, perceived sleep quality (*p* = 0.34) and feeling rested after waking (*p* = 0.67) did not differ significantly.

Ito‐Masui et al. [15] provided wearable‐based sleep measurements and found no significant difference in total time in bed between the 12‐ and 8‐h cohorts (418.5 vs. 398.0 min; adjusted *p* = 1.000). Sleep efficiency also did not differ significantly. The 8‐h cohort had more daily sleep episodes, although this difference was not significant after adjustment (adjusted *p* = 0.083). At the sleep‐episode level, short sleep episodes before and after night shifts were significantly more frequent in the 8‐h cohort (adjusted *p* ≤ 0.001). However, total time in bed after a night shift was significantly longer in the 8‐h cohort (adjusted *p* = 0.001).

### 2.6. Anxiety and Psychological Health

Findings for anxiety and psychological health did not show a consistent advantage for either shift pattern. Iskera‐Golec et al. [11] found significantly higher cognitive anxiety among nurses working 12‐h shifts (16.16 vs. 13.48, *p* < 0.05). No significant differences were found for somatic anxiety or general psychological ill‐health. In contrast, Dwyer et al. [19] reported that participants perceived less stress related to time management under the 12‐h roster (*M* = 3.58, SD = 0.79). In addition, Ito‐Masui et al. [15] reported lower daily self‐rated stress in the 12‐h cohort, but the difference was not statistically significant after Bonferroni correction for multiple comparisons (adjusted *p* = 0.208).

### 2.7. Sick Leave

Objective findings for sick leave varied across the included studies. Webster et al. [16] reported a reduction of 69 sick‐leave days during the 12‐h roster period compared with the earlier 8 h period. Similarly, sick‐leave hours per 1000 h worked were lower during the 12‐h roster period (34.3 vs. 39.8, *p* < 0.0001). In contrast, Battle and Temblett [12] found no significant difference in sickness rates between the 8‐h roster period and the subsequent period in which 12‐h shifts were introduced (9.0 vs. 9.8, *p* = 0.29). Dwyer et al. [19] reported that participants perceived a reduction in sick leave following introduction of the 12‐h roster (*M* = 3.75, SD = 0.45), although this finding was based on participants’ perceptions rather than organisational records.

### 2.8. Well‐Being

#### 2.8.1. Burnout

Battle and Temblett [12] reported lower emotional exhaustion after the introduction of 12‐h shifts than during the preceding 8‐h roster period (17.3 vs. 28.4, *p* = 0.001). A similar pattern was observed for depersonalisation (4.2 vs. 7.6, *p* = 0.002), whereas personal accomplishment did not differ significantly between periods (37.3 vs. 34.1, *p* = 0.541).

In contrast, Iskera‐Golec et al. [11] reported significantly higher emotional exhaustion among nurses working 12‐h shifts than among those working 8‐h shifts (21.37 vs. 16.80, *p* < 0.05). Differences in depersonalisation (7.43 vs. 9.27) and personal accomplishment (28.39 vs. 31.00) were not statistically significant.

#### 2.8.2. Subjective Well‐Being

Findings for subjective well‐being also differed between studies. Dwyer et al. [19] reported that participants agreed that their overall psychological well‐being had improved after the introduction of the 12‐h roster (*M* = 3.82, SD = 0.75) on a 5‐point agreement scale. Participants also reported improvement in physical well‐being (*M* = 3.73, SD = 0.78). Ito‐Masui et al. [15] reported higher median morning well‐being scores in the 12‐h cohort than in the 8‐h cohort (250.0 vs. 222.5) using a digital visual analogue scale, with total scores ranging from 0 to 500. Evening scores were also higher (250.0 vs. 226.0). However, neither difference was statistically significant after adjustment for multiple comparisons (adjusted *p* = 0.07 and 0.16, respectively).

### 2.9. Job Satisfaction

#### 2.9.1. Job and Roster Satisfaction

Findings for job and roster satisfaction varied according to the outcome measured and the method of assessment. Webster et al. [16] reported that roster satisfaction increased from 29.6% during the 8‐h roster to 99.2% after the 12‐h roster was introduced (risk ratio = 3.36, 95% confidence interval [2.62, 4.28]).

Dwyer et al. [19] also reported greater satisfaction with the 12‐h roster with 58% of participants indicating increased satisfaction at work and 92% expressing satisfaction with the 12‐h roster. These findings indicate positive perceptions of the roster but do not establish whether the schedule improved overall job satisfaction relative to an 8‐h roster.

By contrast, Iskera‐Golec et al. [11] found no statistically significant difference in general job satisfaction between nurses working 12 and 8‐h shifts (21.44 vs. 21.81). Although nurses reported high satisfaction with the 12‐h roster itself, this did not translate into a measurable improvement in general job satisfaction assessed by a validated instrument.

### 2.10. Work–Life Balance and Free Time

Findings for work–life balance and free time generally indicated more positive perception under 12‐h schedules. Webster et al. [16] reported that 80% of nurses experienced a difficult in attending sporting, social, or family activities during the 8‐h roster, compared with 54.2% after the introduction of the 12‐h roster. In addition, 89.6% believed that a 12‐h roster would improve work–life balance. Among respondents reporting benefits during the second phase, 98.7% identified improved work–life balance.

Dwyer et al. [19] reported a similar pattern, with most nurses working the 12‐h roster describing greater flexibility in their personal lives. Specifically, 92% reported greater personal flexibility, 92% had more time for social activities, and 83% reported having more time to spend with family.

Iskera‐Golec et al. [11] found significantly greater satisfaction with available free time among nurses working 12‐h shifts than among those working 8‐h shifts (55.45 vs. 39.77, *p* < 0.001). However, general job satisfaction did not differ significantly between groups. Overall, the evidence suggests that the perceived benefits of 12‐h schedules were more consistent for time outside work and work–life balance than for general job satisfaction.

### 2.11. Certainty of Evidence

The certainty of evidence across the assessed outcomes ranged from low to very low (Table 6). Evidence was rated as low certainty for sick leave and work–life balance/free‐time satisfaction outcomes. Very low certainty was assigned to fatigue, sleep, anxiety and psychological health, burnout, well‐being, roster satisfaction and general job satisfaction, primarily due to inconsistency in findings across studies and the limited evidence base, with many outcomes reported by only one or two studies.

**TABLE 6:** GRADE certainty of evidence for synthesised outcomes.

Outcome	Contributing studies	Risk of bias	Inconsistency	Indirectness	Imprecision	Publication bias	Certainty
Fatigue	Iskera‐Golec et al. [11], Dwyer et al. [19], Webster et al. [16], Ito‐Masui et al. [15] (*n* = 4)	Serious	Serious	Not serious	Not serious	Not strongly suspected	**Very low ⨁◯◯◯**
Sleep	Iskera‐Golec et al. [11], Dwyer et al. [19], Webster et al. [16], Ito‐Masui et al. [15] (*n* = 4)	Not serious	Serious	Not serious	Not serious	Not strongly suspected	**Very low ⨁◯◯◯**
Anxiety/psychological health	Iskera‐Golec et al. [11], Dwyer et al. [19], Ito‐Masui et al. [15] (*n* = 3)	Not serious	Serious	Not serious	Not serious	Not strongly suspected	**Very low ⨁◯◯◯**
Sick leave	Dwyer et al. [19], Battle and Temblett [12], Webster et al. [16] (*n* = 3)	Not serious	Not serious	Not serious	Not serious	Not strongly suspected	**Low ⨁⨁◯◯**
Burnout	Iskera‐Golec et al. [11], Battle and Temblett [12] (*n* = 2)	Serious	Serious	Not serious	Not serious	Not strongly suspected	**Very low ⨁◯◯◯**
Subjective well‐being	Dwyer et al. [19], Ito‐Masui et al. [15] (*n* = 2)	Not serious	Not serious	Not serious	Not serious	Not strongly suspected	**Very low ⨁◯◯◯**
Roster satisfaction	Dwyer et al. [19], Webster et al. [16] (*n* = 2)	Not serious	Not serious	Not serious	Not serious	Not strongly suspected	**Very low ⨁◯◯◯**
General job satisfaction	Iskera‐Golec et al. [11] (*n* = 1)	Not serious	Not serious	Not serious	Not serious	Not strongly suspected	**Very low ⨁◯◯◯**
Work–life balance/free‐time satisfaction	Iskera‐Golec et al. [11], Dwyer et al. [19], Webster et al. [16] (*n* = 3)	Not serious	Not serious	Not serious	Not serious	Not strongly suspected	**Low ⨁⨁◯◯**

*Note:* Bold values indicate the overall assessment of the risk of bias.

## 3. Discussion

This systematic review found no consistent evidence that either 12‐ or 8‐h shifts were superior for ICU nurses across the outcomes examined. In this review, nurses tended to report more favourable perceptions of 12‐h rosters in relation to roster satisfaction, flexibility and work–life balance, while findings for fatigue, sleep, psychological health and burnout were less consistent. These findings are consistent with the wider nursing literature, where evidence regarding the effects of 12‐h shifts on ICU nurses’ physical and mental well‐being and job satisfaction remains mixed. [5]. For clinical practice and workforce planning, this distinction is important because a roster that nurses prefer may not necessarily provide better protection against fatigue or poorer health outcomes. Favourable perceptions of 12‐h rosters should therefore not be interpreted as evidence of improved health or well‐being.

The mixed findings for nurse health and well‐being suggest that shift duration should not be considered in isolation from the broader roster context. One possible explanation for the inconsistent results is that changes in shift length are often accompanied by changes to other scheduling characteristics, including the frequency of night shifts, the number of consecutive shifts worked, opportunities for rest and recovery between shifts, the number of days off, and the occurrence of quick returns between shifts [23–25]. Consequently, any observed effects may reflect the combined influence of multiple roster features rather than shift duration alone [25, 26]. While 12‐h rosters may provide advantages such as fewer working days and longer periods away from work, these benefits may be offset by increased fatigue and reduced performance associated with working for extended continuous periods [23, 25]. This highlights the importance of considering roster design as a whole when evaluating the impact of shift patterns on nurse well‐being [24, 25].

Although 12‐h schedules provided nurses with more time away from work, our findings suggest they were associated with less favourable outcomes for fatigue, sleep and emotional exhaustion. This suggests that additional time away from work may not necessarily compensate for the demands of longer shifts. Fatigue among nurses working 12‐h shifts has been associated with shift patterns and individual factors [27]. Poorer sleep, greater fatigue and limited recovery between shifts have also been associated with decision regret among critical‐care nurses [28]. Ferreira et al. [29] reported changes in sleepiness and fatigue over 12‐h NICU shifts. Although these studies did not directly compare 12‐ and 8‐h shifts, they suggest that fatigue and recovery may depend on how shifts are organised as well as their duration. For nursing management, this means that changing shift length alone may be insufficient. Night work, consecutive shifts, overtime and recovery time also need to be considered when 12‐h rosters are planned.

In this review we found that nurses tended to view 12‐h schedules favourably in terms of roster preference, flexibility and free time. However, these positive perceptions were not consistently accompanied by better overall job satisfaction or well‐being. In a large cross‐sectional study of hospital nurses across 12 European countries, those working shifts of 12 h or longer were more likely to report burnout, job dissatisfaction and an intention to leave than those working shifts of 8 h or less [4]. Although this study was not ICU‐specific, it shows that preference for a particular roster does not necessarily reflect better occupational well‐being or a lower intention to leave.

The nature of the included evidence also needs to be considered. Several studies evaluated a change in roster or compared different roster periods rather than concurrently comparing nurses working 12‐ and 8‐h shifts. In addition, some outcomes relied on nurses’ perceptions rather than validated measures or objective data. These methodological limitations reduced confidence in the findings and contributed to the low or very low certainty of evidence. Some studies used routine incident data to assess the clinical safety of 8‐ and 12‐h shift schedules. However, these findings should be interpreted cautiously, as similar numbers of reported incidents do not necessarily indicate equivalent levels of safety. Incident‐reporting systems may not capture all fatigue‐related errors or near misses. A systematic review of nurses in acute‐care hospitals also found that shifts of 12 h or longer may be associated with a higher risk of error than shorter shifts, although the evidence was heterogeneous and not ICU‐specific [13]. These findings suggest that shift duration should be considered within the broader roster structure in ICU workforce planning, rather than as an isolated scheduling decision.

Since completion of this review, Tomé and Javier [30] published a descriptive systematic review examining the impact of 12‐h shifts in intensive care nursing. While both reviews address shift duration in ICU settings, their objectives and methodologies differ substantially. Berrocal Tomé synthesised a broad body of evidence relating to 12‐h shifts, including observational studies, qualitative research and reviews addressing fatigue, burnout, patient safety, organisational factors and well‐being. In contrast, the present review focused specifically on studies directly comparing 12‐ and 8‐h shifts among ICU nurses and applied formal methodological quality appraisal and GRADE assessment. Consequently, overlap between the reviews was limited to a small number of primary studies [12, 16], with much of the evidence included by Tomé and Javier [30] comprising noncomparative [27, 29] or broader shift‐work literature [31]. Despite these methodological differences, both reviews reached broadly similar conclusions: Evidence regarding the benefits and harms of 12‐h shifts remains mixed and context dependent. The additional contribution of the present review is the identification that, when restricted to direct comparisons between 12‐ and 8‐h shifts in ICU nurses, the certainty of evidence is low to very low and does not consistently favour either shift model.

## 4. Implications for Nursing Management

The current evidence does not support recommending either 12‐ or 8‐h shifts for all ICU settings. Shift duration should be considered alongside other aspects of the roster, including night work, consecutive shifts, recovery time, overtime, workload, staffing and staff preferences. When roster changes are introduced, nurse managers should consider staff experiences alongside validated health, well‐being, workforce and safety measures. This may help determine whether a particular roster is suitable for the local ICU setting.

### 4.1. Limitations and Research Priorities

This review has several limitations. First, only five studies met the inclusion criteria limiting the strength and generalisability of the conclusions. The small evidence base, together with heterogeneity in study designs, outcome measures and reported findings, limited the ability to draw firm conclusions or determine whether one shift pattern consistently provides greater benefits than the other. Second, most of the included studies were observational or assessed outcomes before and after roster changes and none used randomised comparisons. These designs limit the ability to determine whether observed differences were attributable to shift duration itself. Third, the potential for confounding could not be excluded, as differences in shift duration were often accompanied by other roster characteristics, such as night work, recovery time between shifts and days off, as well as contextual factors including staffing and workload. The independent effect of shift duration could therefore not be clearly separated from these factors. Fourth, several outcomes were based on nurses’ own perceptions rather than validated or objective measures. Thus, perceived benefits may not reflect actual changes in health, well‐being, or retention. The absence of reported differences should therefore be interpreted cautiously. Finally, publication bias could not be formally assessed because of the small number of included studies and the absence of a quantitative meta‐analysis.

## 5. Conclusion

This review found no clear overall difference between 12‐ and 8‐h shifts for ICU nurses across the outcomes examined. The certainty of the available evidence ranged from low to very low. The main benefits reported for 12‐h shifts were greater roster satisfaction, more flexibility, better work–life balance and greater satisfaction with time away from work. Findings for fatigue, sleep, psychological well‐being, burnout, sick leave and overall job satisfaction were mixed. The evidence was limited by differences between studies, variation in roster patterns and the use of self‐reported outcomes. Therefore, shift length should be considered alongside the wider roster pattern. Further research using stronger comparative methods and consistent outcome measures is needed to compare 12‐ and 8‐h shifts among ICU nurses.

**Nomenclature**
ICU: Intensive care unit
PICOs: Population, Intervention, Comparison, Outcome
PRISMA: Preferred Reporting Items for Systematic Reviews and Meta‐Analyses
PROSPERO: International Prospective Register of Systematic Reviews
JBI: Joanna Briggs Institute
MMAT: Mixed Methods Appraisal Tool
GRADE: Grading of Recommendations Assessment, Development and Evaluation

## Funding

This project was not funded. Open access publishing facilitated by Monash University, as part of the Wiley ‐ Monash University agreement via the Council of Australasian University Librarians.

## Conflicts of Interest

The authors declare no conflicts of interest.

## Supporting Information

Additional supporting information can be found online in the Supporting Information section.

## Supporting information


**Supporting Information** PRISMA‐2020 checklist.

## Data Availability

The data supporting the findings of this study are available within the article.

## Appendix A

**TABLE A1:** Ovid MEDLINE search strategy (executed on 21 June 2026).

Search step	Query
1	((ICU or intensive or critical or acute or emergency or trauma) adj3 nurs^∗^).mp. [mp = title, book title, abstract, original title, name of substance word, subject heading word, floating subheading word, keyword heading word, organism supplementary concept word, protocol supplementary concept word, rare disease supplementary concept word, unique identifier, synonyms, population supplementary concept word, anatomy supplementary concept word]
2	Nurse Specialists/
3	1 or 2
4	((‘8’ or ‘12’ or twelve or eight or standard or extended or long) adj3 shift?).mp. [mp = title, book title, abstract, original title, name of substance word, subject heading word, floating subheading word, keyword heading word, organism supplementary concept word, protocol supplementary concept word, rare disease supplementary concept word, unique identifier, synonyms, population supplementary concept word, anatomy supplementary concept word]
5	shift work.mp.
6	Shift Work Schedule/
7	4 or 5 or 6
8	3 and 7

**TABLE A2:** CINAHL search strategy (executed on 21 June 2026).

Search step	Query
1	(ICU OR intensive OR critical OR acute OR trauma OR emergency) N5 nurs^∗^
2	MH ‘Nurse Specialists’
3	S1 or S2
4	((‘8’ OR ‘12’ OR twelve OR eight OR standard OR extended OR long) N5 shift)
5	‘shift work’
6	MH ‘Shift Work Schedule+’
7	S4 or S5 or S6
8	S3 and S7

**TABLE A3:** EMBASE search strategy (executed on 21 June 2026).

Search step	Query
1	((ICU or intensive or critical or acute or trauma) adj3 nurs^∗^).mp. [mp = title, abstract, heading word, drug trade name, original title, device manufacturer, drug manufacturer, device trade name, keyword heading word, floating subheading word, candidate term word]
2	Nurse Specialists/
3	1 or 2
4	((‘8’ or ‘12’ or twelve or eight or standard or extended or long) adj3 shift).mp. [mp = title, abstract, heading word, drug trade name, original title, device manufacturer, drug manufacturer, device trade name, keyword heading word, floating subheading word, candidate term word]
5	shift work.mp.
6	Shift Work Schedule/
7	4 or 5 or 6
8	3 and 7

**TABLE A4:** Web of Science search strategy (executed on 21 June 2026).

Search step	Query
1	TS = ((ICU OR intensive OR critical OR acute OR emergency OR trauma) NEAR/3 nurs^∗^)
2	TS= (‘nurse specialist^∗^’ OR ‘clinical nurse specialist^∗^’ OR ‘specialist nurse^∗^’)
3	1 OR 2
4	TS = ((8 OR 12 OR eight OR twelve OR standard OR extended OR long) NEAR/3 shift^∗^)
5	TS = (‘shift work’)
6	4 OR 5
7	3 AND 6

**TABLE A5:** Scopus search strategy (executed on 21 June 2026).

Search step	Query
1	TITLE‐ABS‐KEY ((ICU OR intensive OR critical OR acute OR emergency OR trauma) W/3 nurs^∗^)
2	TITLE‐ABS‐KEY (‘nurse specialist^∗^’ OR ‘clinical nurse specialist^∗^’ OR ‘specialist nurse^∗^’)
3	#1 OR #2
4	TITLE‐ABS‐KEY ((8 OR 12 OR eight OR twelve OR standard OR extended OR long) W/3 shift^∗^)
5	TITLE‐ABS‐KEY (‘shift work’)
6	4 OR 5
7	3 AND 6
